# Switch in FOXA1 Status Associates with Endometrial Cancer Progression

**DOI:** 10.1371/journal.pone.0098069

**Published:** 2014-05-21

**Authors:** Ingvild Løberg Tangen, Camilla Krakstad, Mari K. Halle, Henrica M. J. Werner, Anne M. Øyan, Kanthida Kusonmano, Kjell Petersen, Karl Henning Kalland, Lars A. Akslen, Jone Trovik, Antoni Hurtado, Helga B. Salvesen

**Affiliations:** 1 Center for Cancer Biomarkers, Department of Clinical Science, University of Bergen, Bergen, Norway; 2 Department of Gynecology and Obstetrics, Haukeland University Hospital, Bergen, Norway; 3 Department of Microbiology, Haukeland University Hospital, Bergen, Norway; 4 Computational Biology Unit, University of Bergen, Bergen, Norway; 5 Center for Cancer Biomarkers, Department of Clinical Medicine, University of Bergen, Bergen, Norway; 6 Department of Pathology, Haukeland University Hospital, Bergen, Norway; 7 Breast Cancer Research group, Centre for Molecular Medicine Norway, University of Oslo, Oslo, Norway; University of Kentucky College of Medicine, United States of America

## Abstract

**Background:**

The transcription factor Forkhead box A1 (FOXA1) is suggested to be important in hormone dependent cancers, although with little data for endometrial cancer. We investigated expression levels of FOXA1 in primary and metastatic endometrial cancer in relation to clinical phenotype, and transcriptional alterations related to FOXA1 status.

**Methods:**

Protein expression of FOXA1 was explored by immunohistochemistry in 529 primary and 199 metastatic endometrial carcinoma lesions. mRNA levels from corresponding 158 fresh frozen primary and 42 metastatic lesions were analyzed using Agilent Microarrays (44k) in parallel.

**Results:**

Low FOXA1 protein expression in primary tumors significantly correlated with low FOXA1 mRNA, high age, non-endometrioid histology, high grade, loss of ERα and PR and poor survival (all p-values <0.05). Through a Connectivity Map search, HDAC inhibitors were suggested as potential treatment for patients with low FOXA1 expression. An increase in FOXA1 expression was observed from primary to metastatic lesions and it correlated with CDKN2A expression in metastases.

**Conclusion:**

Low FOXA1 is associated with poor survival and suggests a potential for HDAC inhibitors in endometrial carcinoma. A switch in FOXA1 expression from primary to metastatic lesions is observed and gene expression indicates a link between FOXA1 and CDKN2A in metastatic lesions.

## Introduction

In the Western world endometrial cancer is the most common gynecological malignancy, and the incidence is rising [1]. Endometrial cancers are broadly classified in two groups. Type I endometrial cancer is most common and is characterized by favorable outcome, endometrioid histology, low stage and grade and often intact expression of hormone receptors. Type II endometrial cancer is associated with poor outcome, non-endometrioid histology, high stage and grade, and has usually lost expression of hormone receptors [2]. Patients are standardly surgically treated with hysterectomy with bilateral salpingoophorectomy with or without lymphadenectomy. The effect of adjuvant systemic treatment is less studied for endometrial compared to ovarian cancer, although similar platinum based chemotherapy regimens in combination with paclitaxel often is used in the adjuvant and systemic disease setting.

Estrogen dependent endometrial cancers are thought to arise from prolonged unopposed exposure to estrogens. Estrogen dependent activation of estrogen receptor α (ERα) has been reported to lead to proliferation through upregulation of growth factors such as epidermal growth factor (EGF) [3], its receptor EGFR [4], insulin like growth factor (IGF-1) [5] and growth enhancing proto-oncogenes like c-myc [6]. Regulation of ERα activity is also known to involve several cofactors including both coactivators and corepressors. In addition the pioneer factor Forkhead box A1 (FOXA1) has been shown to be an important regulator of ERα activity through facilitating binding of ERα [7].

FOXA1 is a member of the Forkhead Box transcription factor family, formerly known as Hepatocyte Nuclear Factor (HNF) family. FOXA1 proteins bind DNA and induce nucleosomal rearrangement that often results in an open chromatin structure [8], [9]. This facilitates the binding of additional transcription factors, including ERα [7]. FOXA1 has been found to be recruited to almost half of all ER binding regions [7].

The association between hormone receptors and known prognostic variables such as FIGO stage, histologic grade and survival has been well documented in endometrial carcinoma [10]–[12]. More knowledge regarding molecular mechanisms involved in estrogen signaling and estrogen-related cofactors in hormone related cancers is needed to develop new therapeutic strategies. Several studies have suggested a role for FOXA1 in different hormone dependent cancers [13]. High FOXA1 expression is correlated with good prognosis in ER positive breast cancer but in prostate cancer FOXA1 level has been associated with either good or bad prognosis depending on the patient group, and has been proposed as a context dependent marker for survival in hormone dependent cancers [14]–[17].

Furthermore, the association between FOXA1 and ER in breast cancer and FOXA1 and androgen receptor (AR) in prostate cancer suggests that expression levels of FOXA1 may influence responsiveness to antihormonal treatment in hormone dependent cancers. On this background, we investigated the expression level of FOXA1 in endometrial cancer in relation to phenotype and established biomarkers including hormone receptor status; and subsequently explore transcriptional alterations related to FOXA1 protein levels in primary and metastatic endometrial carcinoma lesions.

## Materials and Methods

### Ethics statement

All parts of the study have been approved according to Norwegian legislation. The study was approved by the Norwegian Data Inspectorate, Norwegian Social Sciences Data Services and the Regional Committee for Medical Research Ethics, REC West (NSD15501; REK 052.01). All participants gave written informed consent.

### Patient series

A population based patient series, including 529 patients diagnosed with endometrial cancer in Hordaland County (Norway) during the period from 2001–2011, was studied. Tissue from primary tumors was included prospectively from consented patients surgically staged according to the International Federation of Gynaecology and Obstetrics (FIGO) 2009 criteria. The clinicopathological variables, age at diagnosis, FIGO stage, histological subtype and grade, treatment and follow-up were collected by review of medical records as reported earlier [18]. For 91 patients with advanced or recurrent disease, biopsies were available from metastatic tissue (a total of 199 FFPE lesions). Fresh frozen tissue (158 primary and 42 metastatic lesions) was collected and prepared in parallel with the formalin-fixed paraffin embedded tissues (FFPE) when available.

### Tissue microarrays

Tissue microarrays (TMAs) were prepared from FFPE tissue from primary tumors and metastases. The area of highest tumor grade was identified on hematoxylin and eosin stained slides. Three tissue cylinders from primary tumors, and one tissue cylinder from metastases (0.6 mm) were punched out from selected areas, and mounted in a recipient paraffin block using a custom made precision instrument (Beecher instruments, Silver Spring, MD, USA). This method for producing TMAs has previously been described and validated [19].

### Immunohistochemistry

Five µm thick TMA sections were dewaxed with xylene and rehydrated in graded ethanol series. For FOXA1 detection, antigen retrieval was performed by microwave boiling for 20 minutes in citrate buffer (pH 6). Endogenous peroxidase and nonspecific binding of primary antibody were blocked with peroxidase block (S2023, Dako, Denmark) and serum free protein block (X0909, Dako, Denmark). Slides were incubated with anti-FOXA1 primary antibody (ab23738, Abcam, UK), diluted 1∶800, for 30 minutes at room temperature. Secondary antibody, combined anti-mouse and anti-rabbit (K5007, Dako, Denmark), was applied for 30 minutes, followed by 8 minutes with Diaminobenzidine (DAB+, K4007, Dako, Denmark) before counterstaining with hematoxylin. Immunohistochemical staining of ERα has previously been described for 477 primary tumors and 78 metastases. Additional 92 primary tumors were stained according to the method reported previously [12].

### Evaluation of staining

IHC staining was evaluated using a semiquantitative system where both intensity of the staining, and area of tumor cells with positive staining were considered. Staining intensity was graded from 0 (no staining) to 3 (strong staining). Proportion of stained tumor cells was graded as 0, 1 (<10%), 2 (10% to 50%) and 3 (>50%). A staining index was calculated as the product of intensity and staining area. In the statistical analysis, staining indexes for FOXA1 were categorized in tertiles considering the frequency distribution for the marker, size of subgroups and the number of events in each category. The two lower tertiles, showing similar survival estimates, were subsequently merged. Cut-off value for ERα was applied as previously defined [12]. To evaluate the reproducibility, two independent observers (ILT and CK) scored random TMA slides, giving κ-value of 0.80 for FOXA1 and 0.82 for ERα [12]. When evaluating heterogenic multiple metastatic lesions form the same patient, FOXA1 was defined as low if any of the metastatic lesions demonstrated low expression.

### Microarray analysis

Freshly frozen tissues from 158 primary tumors and 42 metastases were collected in parallel with the FFPE tissues. Extraction of RNA was done using the RNeasy Mini Kit (Qiagen, Hilden, Germany). RNA was hybridized to Agilent Whole Human Genome Microarrays 44k (Cat. no. G4112F) according to the manufacturer's instruction, and the arrays were scanned using the Agilent Microarray Scanner Bundle. The J-express software (Molmine, Bergen, Norway) was used to analyze the data. Median spot signal was used as intensity measure and expression data were normalized using quantile normalization. Microarray data have been deposited in the ArrayExpress Archive database, http://www.ebi.ac.uk/arrayexpress/ with the Accession Number E-MTAB-2532. Genes differentially expressed between groups were identified by SAM (Significance Analysis of Microarray) analysis and were considered significant if False Discovery Rate (FDR)<0.01. If the number of samples did not allow the use of SAM, FSS (Feature Subset Selection) analysis was used and P<0.01 was considered significant.

### Connectivity Map

Correlation between gene expression and potential therapeutics was investigated using the drugs signature database Connectivity Map [20]. The drug signatures in this database is generated by genes differentially expressed in cell lines treated with drugs compared to untreated cell lines, and reflect the genes presumed to be altered by the drug. Genes differentially expressed according to FOXA1 status, both within the whole patient population and within the ERα negative group were the basis for analysis in Connectivity Map.

### Statistical analysis

The software package SPSS 19.0 (SPSS Inc, Chicago, IL) was used for statistical analyses. Statistical significance was defined as probability <0.05. Association between groups was evaluated using the Pearson χ^2^ test for categorical variables. Univariate survival analysis was performed using the Kaplan-Meier (product-limit) method with date of primary surgery as entry date and time to death due to endometrial carcinoma as endpoint (disease specific survival). Patients who died from other causes were censored at the date of death. The log-rank test (Mantel-Cox) was used to compare survival between groups. The Cox proportional hazard regression model was used to evaluate the prognostic impact of FOXA1 adjusted for other prognostic markers in endometrial cancer. Due to an interaction between FIGO stage and histologic type, Cox analysis was performed in the endometrioid subtype.

## Results

### Low FOXA1 expression is associated with poor prognosis in endometrial cancer

**Figure 1:**
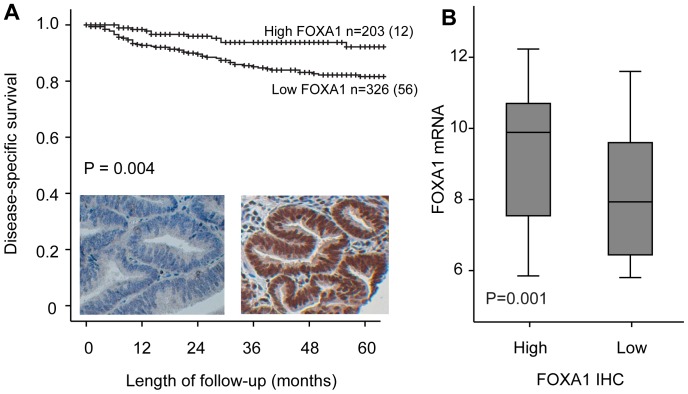
FOXA1 and survival. (A) Kaplan-Meier curve showing disease specific survival according to FOXA1 protein level. Low FOXA1 expression is significantly correlated with reduced survival for patients with endometrial carcinoma. Left and right insert shows low and high nuclear expression of FOXA1 respectively. (B) Low protein expression of FOXA1 is significantly correlated with low FOXA1 mRNA expression.

**Figure 2:**
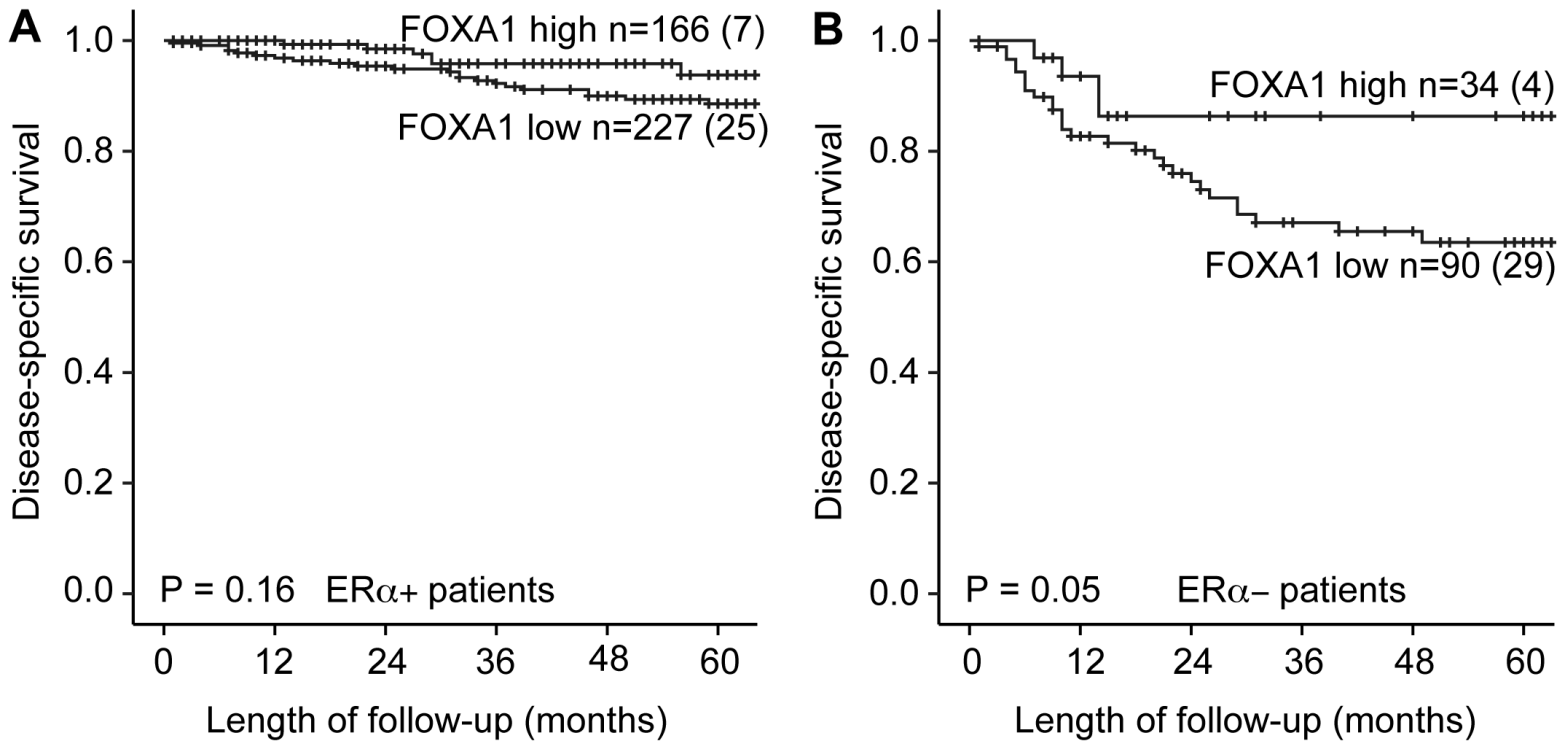
FOXA1 survival analyses stratified for ERα status in the tumors. (A) Low FOXA1 expression among ERα positive patients did not significantly impact survival. (B) Amongst ERα negative patients FOXA1 expression significantly influenced survival with worst prognosis for patients with low FOXA1.

FOXA1 has previously been shown to be a pioneer factor for ER in breast cancer (Figure S1). We investigated a large and unique collection of endometrial cancer samples to elucidate a potential similar role for FOXA1 in these diseases. Protein expression of FOXA1 was primarily nuclear. The protein expression was seen both in stromal and glandular tissues, but only staining in the nuclei of glandular tissue was scored. When comparing FOXA1 status with available clinicopathological variables, low FOXA1 expression (score index from 0–4) was significantly correlated with high age (P = 0.04), non-endometrioid histology (P = 0.002), high histologic grade (P = 0.003), loss of PR (P = 0.02) and ERα (P = 0.003) (Table 1). Low FOXA1 protein expression was significantly associated with reduced disease specific survival (P = 0.004) (Figure 1A) and low FOXA1 mRNA expression (P = 0.001) (Figure 1B). For patients with ERα positive tumors, we found no impact of FOXA1 (Figure 2A) in contrast to the ERα negative group where low FOXA1 expression was significantly associated with worse outcome (Figure 2B). In a multivariate analysis, in the endometrioid subgroup, using the Cox proportional hazard model with age, FIGO stage, histologic grade, FOXA1 and ERα expression as variables, FOXA1 expression was found to have prognostic impact similar to grade and ERα status, although with no independent prognostic impact (Table S1).

**Table 1:** FOXA1 protein expression in 529 patients with endometrial carcinoma related to clinicopathologic variables

	FOXA1		
Variable	High n (%)	Low n (%)	*P*-value
Age			0.04
<66	118 (42)	160 (58)	
≥66	85 (34)	166 (66)	
FIGO-09 stage			0.06
I–II	176 (40)	262 (60)	
III–IV	27 (30)	64 (70)	
Histologic type			0.002
Endometrioid*	180 (42)	254 (58)	
Non-endometrioid	23 (24)	72 (76)	
Histologic grade			0.003
Grade 1/2	149 (43)	199 (57)	
Grade 3	52 (29)	125 (70)	
Metastatic nodes			0.07
Negative	165 (43)	215 (57)	
Positive	16 (30)	37 (70)	
Ploidy			0.06
Diploid	111 (40)	170 (60)	
Aneuploid	21 (28)	55 (72)	
ERα			0.003
Positive	166 (42)	227 (58)	
Negative	34 (27)	90 (73)	
**PR**			0.02
Positive	124 (39)	197 (61)	
Negative	29 (27)	80 (73)	

Missing: histologic grade: 4, metastatic nodes: 96, ploidy: 172, ERα: 12, PR: 99.

Abbreviations: ERα = estrogen receptor α; PR = progesterone receptor. P-values based on the Chi-square test. *Endomterioid carcinomas including cases with areas with squamous cell differentiation but no pure squamous cell carcinomas; Non-endometrioid subtype included serous, clear cell, undifferentiated histologies and carcinosarcomas.

### Gene expression related to FOXA1 and ERα expression does not overlap

As for breast cancer, ERα loss has been consistently associated with poor survival in patients with endometrial cancer [2], [11], [12]. As FOXA1 is shown to be important for ERα-chromatin interactions in breast cancer, we further investigated whether FOXA1 related gene expression resembles ERα regulated gene expression in endometrial cancer. SAM analysis identified 468 genes differentially expressed in tumors with intact ERα expression compared to loss, while 506 genes were differentially expressed in tumors with high FOXA1 expression compared to low. Only three overlapping genes were identified in the ERα and FOXA1 related gene lists (Table S2 and S3).

### HDAC inhibitors are suggested as potential drugs for treatment of endometrial cancers with low FOXA1

Genes differentially expressed related to FOXA1 status were used to query Connectivity Map for compounds with potential to revert the gene signature of patients with low FOXA1 status. Two HDAC inhibitors came up among the top five ranked compounds (Table 2). These two HDAC inhibitors were also the two top ranked anticorrelated compounds to low FOXA1 expression within the ERα negative patient subgroup (Table 2) (Table S4).

**Table 2:** Top ranked drugs and targets for therapy related to FOXA1 status, based on Connectivity Map

Drug signatures negatively correlated to endometrial cancer with low FOXA1 expression				
Rank	Name of compound	Description	n^a^	P^b^
1	Semustine	Alkylating agent†	4	<0.001
2	Withaferin A	Angiogenesis inhibitor [31]	4	<0.001
3	Vorinostat	HDAC inhibitor*	12	<0.001
4	Thioridazine	Protein kinase inhibitor*	20	<0.001
5	Trichostatin A	HDAC inhibitor*	182	<0.001
**Drug signatures negatively correlated to ERα negative endometrial cancer with low FOXA1 expression**				
1	Vorinostat	HDAC inhibitor*	12	<0.001
2	Trichostatin A	HDAC inhibitor*	182	<0.001
3	Thioridazine	Protein kinase inhibitor*	20	<0.001
4	Ciclosporin	Immunosuppressive [32]	6	<0.001
5	5707885		4	<0.001

† Function as described Martindale monograph.

*Function as described in ChemBank (http://chembank.broadinstitute.org/).

N^a^ number of instances in which the compounds were tested in Connectivity map.

P^b^ The p-value for each compound represents the distribution of these scores compared with the distribution of scores among all small molecules, using a permutation test as described by Lamb et al [20].

### Metastatic spread is associated with a shift in FOXA1 expression

**Figure 3:**
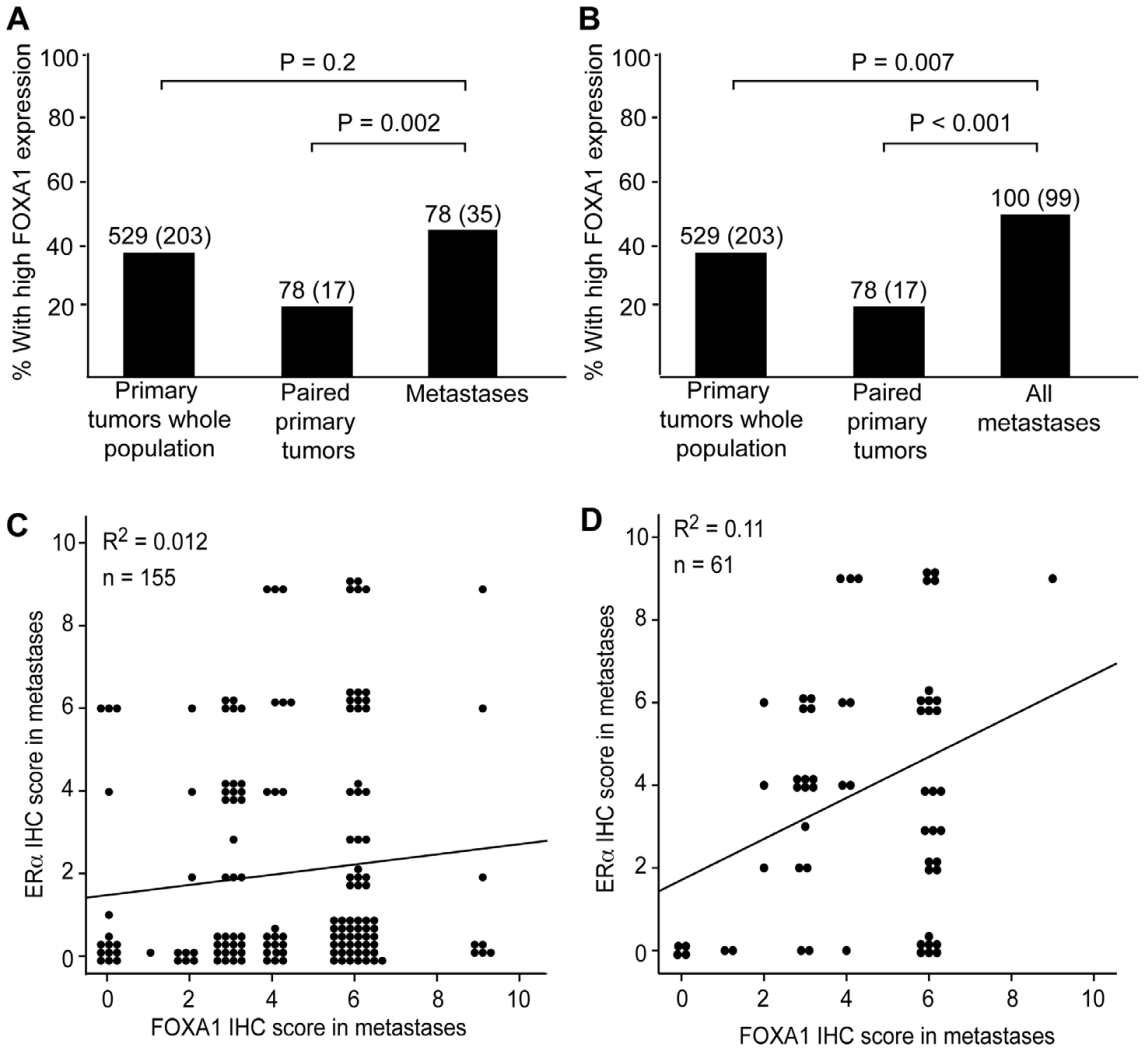
Increased expression of FOXA1 in metastases. (A) FOXA1 expression is retained from primary tumors to metastases, while the expression is significantly increased from paired primary tumors to their corresponding metastases. FOXA1 is defined as low if any of the metastatic lesions from the individual cases explored (n = 78) demonstrated low expression. (B) Looking at all metastases (n = 199) there is a significant increase in expression from primary to metastatic lesions. Numbers indicate number of cases investigated with number of cases with high expression in parenthesis. (C) There is no correlation between FOXA1 and ERα expression in metastases and the correlation between FOXA1 and ERα expression in metastases only from ERα positive primary tumors is low (D).

Metastatic lesions from 91 patients were stained for FOXA1 expression to investigate FOXA1 status in metastatic lesions. When defining FOXA1 as low if any of the metastatic lesions demonstrated low expression, no significant change in expression was seen between the whole population of primary tumors and the metastatic lesions (Figure 3A). Surprisingly, when comparing FOXA1 levels for primary tumors from patients with systemic disease with their metastatic lesion counterparts, expression of FOXA1 was found to be significantly higher in the metastatic lesions compared to the corresponding primary lesion. This was also true when all metastatic lesions, independent of the number of metastases per patient, were examined (Figure 3B). We have previously found that there is a significant loss of expression of ERα from primary to metastatic lesions [12]. When comparing FOXA1 and ERα expression in metastatic lesion no correlation between the expression levels was found (r^2^ = 0.012) (Figure 3C). The same was observed when comparing FOXA1 levels in metastases from only ERα positive primary endometrial tumors (r^2^ = 0.11) (Figure 3D).

### FOXA1 and CDKN2A levels are linked in metastatic lesions

**Figure 4:**
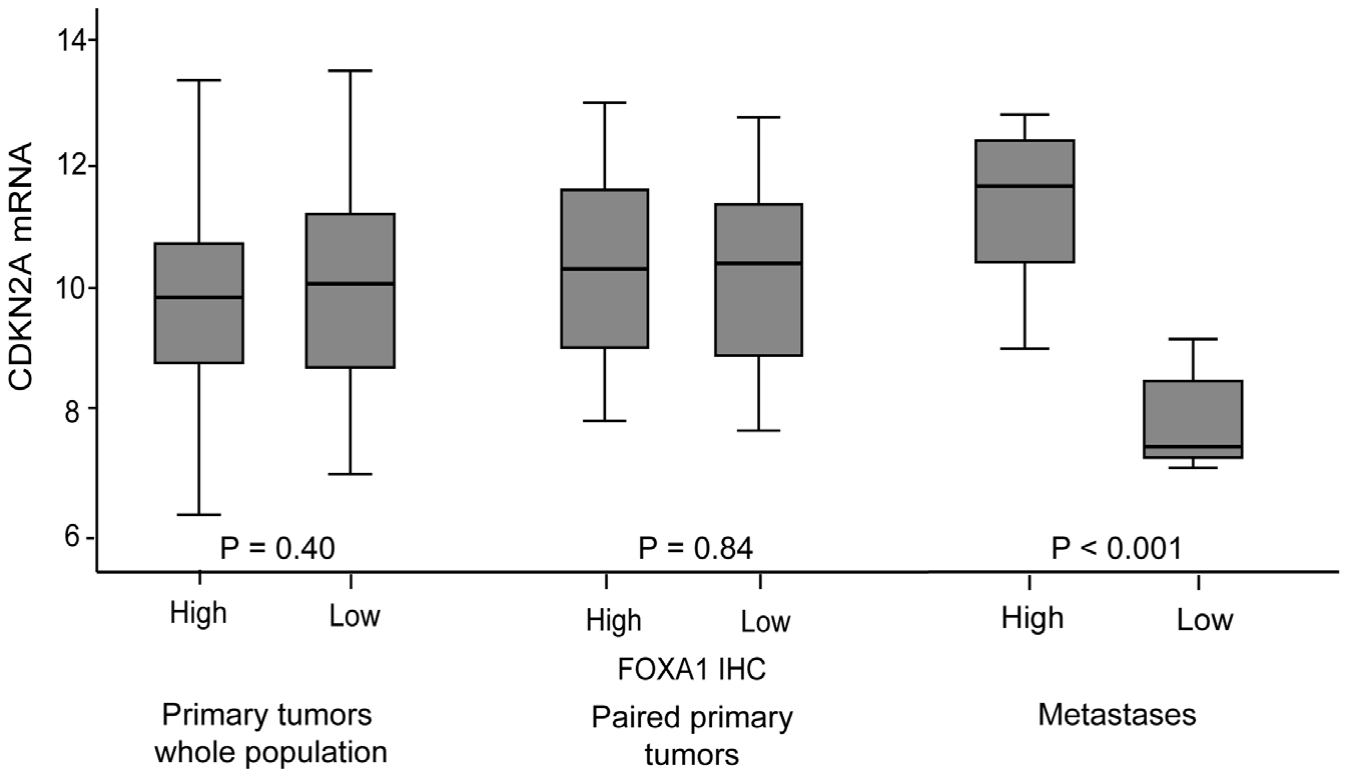
FOXA1 and CDKN2A. Protein expression of FOXA1 is significantly associated with CDKN2A mRNA expression in metastases, but not in primary tumors.

To investigate differences in transcriptional effects related to FOXA1 levels in primary and metastatic lesions we further explored genes differentially expressed according to FOXA1 status in primary tumors compared to metastases (Table S3 and S5). The overlap between the genes differentially expressed was low (only 26 genes). Interestingly, one of the top ranked genes differentially expressed according to FOXA1 protein expression in metastatic lesions was CDKN2A. FOXA1 was recently shown to control CDKN2A expression [21]. To investigate whether this association was limited to the metastatic setting, we investigated a potential link between FOXA1 and CDKN2A also in primary tumors (Figure 4). A significant association between FOXA1 and CDKN2A was only observed in metastatic lesions.

## Discussion

FOXA1 has been recognized as an important transcription factor that modulates the functions of steroid receptors such as estrogen receptor in breast cancer and AR in prostate cancer. FOXA1 as a prognostic marker in breast cancer has been extensively studied [14], [22], and high expression of FOXA1 is correlated with better disease specific survival, ERα positivity and the luminal subtype A. In prostate cancer high expression of FOXA1 predicts poor prognosis, and correlates with AR positivity [15]. Also, the recent publication by The Cancer Genome Atlas (TCGA) Research Network showed low ER/FOXA1 signaling to be associated with poor survival in endometrial cancer [23]. Our findings that low FOXA1 expression is significantly associated with markers for worse outcome as well as poor survival appear to be in line with the TCGA data and findings in breast cancer, but in contrast to prostate cancer and one previous small, and potentially underpowered study of 109 endometrial carcinoma cases showing no significant prognostic impact of FOXA1 expression [24].

In breast cancer cells FOXA1 has been shown to be necessary for estrogen response [7]. Similar to breast cancer, endometrial cancer is an estrogen dependent cancer and type 1 endometrial cancer is associated with expression of ERα. ERα positive patients have a significantly better survival than patients with ERα loss and ERα is a strong prognostic marker in endometrial cancer [2]. Studies in breast cancer have shown that ERα positive patients with high expression of FOXA1 have a significantly better survival compared to patients with low expression of FOXA1 [14], [25]. We could not find a similar strong survival effect for FOXA1 within the ERα positive group, but only within the ERα negative group of patients. If the reduced survival observed for the FOXA1 negative group only was due to loss of ERα a significant association between FOXA1 and survival within the ERα negative group would not have been expected. This could indicate that FOXA1 in endometrial cancer may have a function that is not linked to ERα, and the effect of FOXA1 could be organ specific. FOXA1 has previously been shown to regulate distinct transcriptional programs in cells of different linages [26], and the clinical experience with increased endometrial cancer risk amongst women with ERα positive breast cancers treated with Tamoxifen also supports organ specific differences in the hormone regulation. Further supporting this, gene expression related to FOXA1 levels did not overlap with expression pattern related to ERα. If FOXA1 is important for regulating ERα activity in endometrial cancer, as is seen in breast cancer, an overlap between the differentially expressed genes would be expected. In addition, our data suggest HDAC inhibitors as potential treatment for patients with low FOXA1 expression, both within the whole patient population and within the ERα negative group. This is interesting, as PI3K and mTOR inhibitors recently have been suggested as promising drugs for patients with ERα negative tumors in particular [11]. The present finding suggests that FOXA1 involvement in ERα regulation might be different in endometrial cancer compared to what is found in breast cancer. This appears to be in line with an endometrial cancer cell line study demonstrating that introduction of FOXA1 suppresses both proliferation and migration [24] in contrast to ERα which leads to proliferation. The role of FOXA1 as a pioneer factor for ERα has also been found to differ in various cancer cell types. Recruitment to chromatin was shown to be dependent of FOXA1 in breast cancer cells, however in an osteoblast like cell line (U2OS-ERα) FOXA1 is not expressed and ERα binding is independent of FOXA1 [27]. In endometrial cancer, FOXA1 is important for inhibition of cell proliferation and migration[24], and in prostate cancer cell lines FOXA1 was recently shown to inhibit cell motility and epithelial to mesenchymal transition (EMT) through regulation of the transcription factor SLUG[17]. Little is known regarding bindingpartners for FOXA1 in endometrial cancer, and more work is needed to elucidate this.

Our finding that FOXA1 protein expression increase from primary tumors to metastases, could appear as contradictory to our finding that high FOXA1 associates with good outcome in patients with endometrial cancer. ERα expression loss has been reported to increase from primary to metastatic endometrial carcinoma lesions [12]. The low correlation between ERα and FOXA1 expression in metastatic lesions, suggests that FOXA1 is less critical for ERα function in endometrial cancer compared to what has been observed for breast cancer [28]. In both prostate and breast cancers high expression of FOXA1 in metastatic lesions were found, however with retained expression of AR and ERα respectively [28], [29]. Taken together, this suggests that the role of pioneer factors in regulation of nuclear receptors in hormone dependent cancers is tissue related. In breast cancer FOXA1 seems important for ER mediated transcription, and silencing of FOXA1 leads to inhibition of ER binding and transcriptional activity [30]. In prostate cancer FOXA1 has been found to be a pioneer factor for AR for some binding events, but also a repressor. Change in FOXA1 expression in prostate cancer therefore seems to lead to reprogramming of AR binding events [15].

The higher expression of FOXA1 in metastases compared to their primary tumor counterparts, and the association between protein expression of FOXA1 and CDKN2A mRNA only in metastatic lesions, may indicate a change in the role of FOXA1 during endometrial cancer progression. Metastases with high expression of FOXA1 also have high expression of CDKN2A. CDKN2A is known to encode several distinct proteins, including p16^INK4A^, p15^INK4B^ and p14^ARF^. Interestingly FOXA1 was recently shown to control p16^INK4A^ expression during cellular senescence [21]. Which protein encoded by CDKN2A that is expressed in metastases is however unknown and needs to be further investigated.

FOXA1 seems to have tissue specific roles that also may change during endometrial cancer progression. In addition, it is likely that other unknown factors than FOXA1 is required for regulation of ERα function in endometrial cancer. Identifying more of these will improve our understanding of tissue specific hormone receptor signaling and will be of relevance when developing targeted therapeutics in ERα related malignant disease, including endometrial cancer. Thus, FOXA1 may add clinically relevant information as a biomarker in endometrial cancers and points to a role for HDAC inhibitors for treatment.

## Supporting Information

Figure S1 **Schematic illustration of factors involved in regulation of ER mediated transcription (E2:estradiol).**
(TIF)

Table S1 **Cox analysis of predictors of endometrial cancer specific survival: effects of FIGO stage, age, histologic grade, FOXA1 and ERα expression within the endometrioid subgroup.**
(DOCX)

Table S2 **SAM analysis of ER positive versus ER negative cases (FDR<0.01).**
(XLSX)

Table S3 **SAM analysis of FOXA1 high versus FOXA1 low cases (FDR<0.01).**
(XLSX)

Table S4 **FSS analysis of FOXA1 high versus FOXA1 low cases in ER negative samples (p-value<0.01).**
(XLSX)

Table S5 **FSS analysis of FOXA1 high versus FOXA1 low cases in metastases (p-value<0.01).**
(XLSX)
