# Supplementary figures and images for: Switch in FOXA1 Status Associates with Endometrial Cancer Progression
Source: PLoS One. 2014 May 21;9(5):e98069. doi: 10.1371/journal.pone.0098069 (PMC4029819; doi:10.1371/journal.pone.0098069)

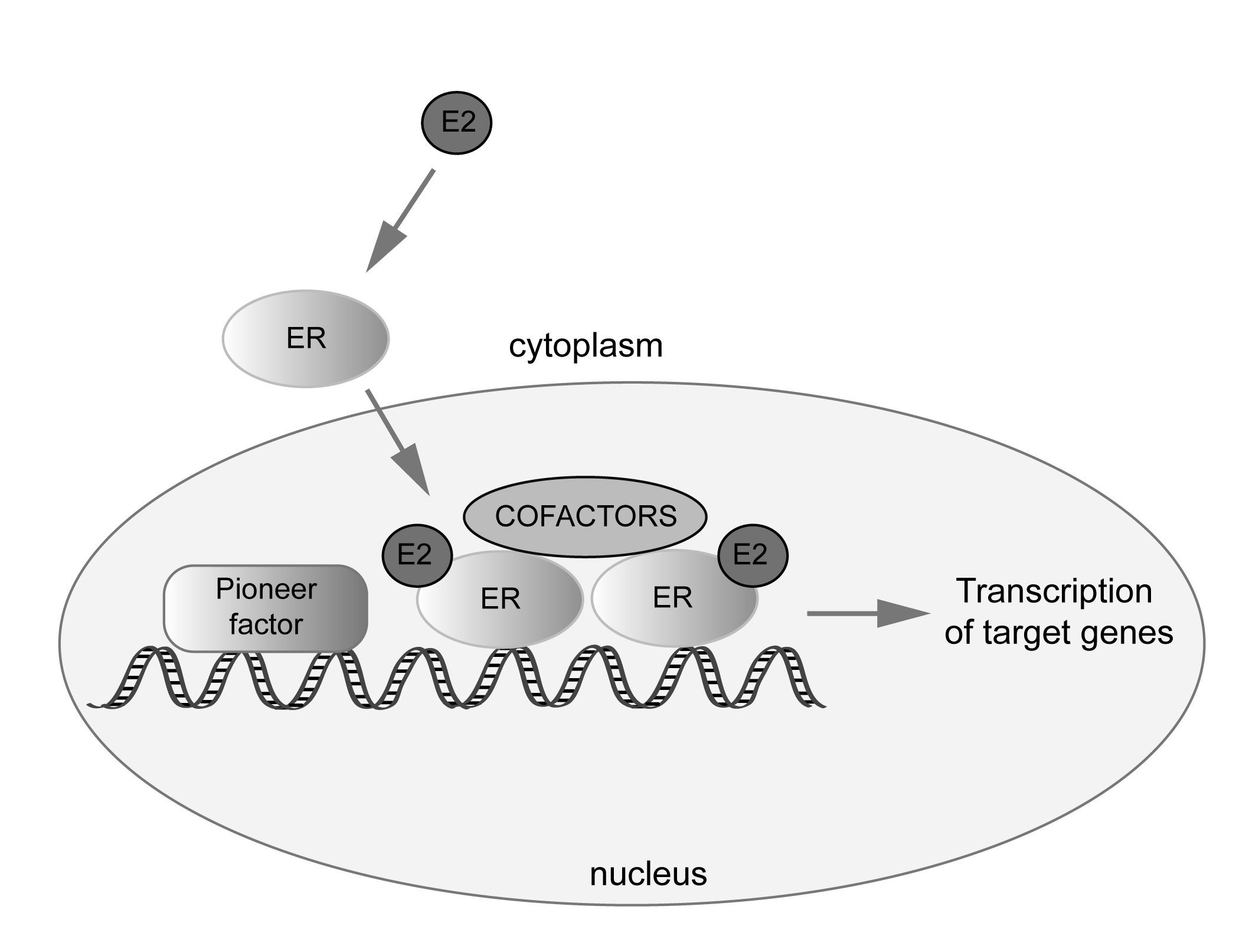

Supplement: Figure S1 — Schematic illustration of factors involved in regulation of ER mediated transcription (E2:estradiol). (TIF) [file pone.0098069.s001.tif]
