# Supplementary material for: Switch in FOXA1 Status Associates with Endometrial Cancer Progression
Source: PLoS One. 2014 May 21;9(5):e98069. doi: 10.1371/journal.pone.0098069 (PMC4029819; doi:10.1371/journal.pone.0098069)
Supplement: Table S1 — Cox analysis of predictors of endometrial cancer specific survival: effects of FIGO stage, age, histologic grade, FOXA1 and ERα expression within the endometrioid subgroup. (DOCX) [file pone.0098069.s002.docx]

Table S1. Cox analysis of predictors of endometrial cancer specific survival: effects of FIGO stage, age, histologic grade, FOXA1 and ERα expression within the endometrioid subgroup.

| **Variable** | **Unadjusted**  **HR** | **95% CI** | **p-value** | **Adjusted**  **HR** | **95% CI** | **p-value** |
| --- | --- | --- | --- | --- | --- | --- |
| Figo stage | 21.88 | 10.5-45.6 | <0.001 | 19.0 | 8.9-40.6 | <0.001 |
| Age | 1.06 | 1.0-1.1 | <0.001 | 1.1 | 1.0-1.1 | 0.003 |
| FOXA1 expression | 1.93 | 0.8-4.5 | 0.13 | 2.0 | 0.8-4.8 | 0.12 |
| ERα expression | 2.57 | 1.2-5.4 | 0.013 | 1.6 | 0.8-3.5 | 0.20 |
| Histologic grade | 3.26 | 1.6-6.6 | 0.001 | 1.6 | 0.8-3,5 | 0.21 |

Unadjusted HR given for analyses of cases with available data for all variables in the multivariate analyses (number of cases: 406, number of events: 33).
